# Aberrant Functional Connectome in Neurologically Asymptomatic Patients with End-Stage Renal Disease

**DOI:** 10.1371/journal.pone.0121085

**Published:** 2015-03-18

**Authors:** Xiaofen Ma, Guihua Jiang, Shumei Li, Jinhui Wang, Wenfeng Zhan, Shaoqing Zeng, Junzhang Tian, Yikai Xu

**Affiliations:** 1 Department of Medical Imaging Center, Nanfang Hospital, Southern Medial University, Guangzhou, PR China; 2 Department of Medical Imaging, Guangdong No. 2 Provincial People’s Hospital, Guangzhou, PR China; 3 Center for Cognition and Brain Disorders, Hangzhou Normal University, Hangzhou, PR China; 4 Zhejiang Key Laboratory for Research in Assessment of Cognitive Impairments, Hangzhou, PR China; Wake Forest School of Medicine, UNITED STATES

## Abstract

**Purpose:**

This study aimed to investigate the topological organization of intrinsic functional brain networks in patients with end-stage renal disease (ESRD).

**Materials and Methods:**

Resting-state functional MRI data were collected from 22 patients with ESRD (16 men, 18–61 years) and 29age- and gender-matched healthy controls (HCs, 19 men, 32–61 years). Whole-brain functional networks were obtained by calculating the interregional correlation of low-frequency fluctuations in spontaneous brain activity among 1,024 parcels that cover the entire cerebrum. Weighted graph-based models were then employed to topologically characterize these networks at different global, modular and nodal levels.

**Results:**

Compared to HCs, the patients exhibited significant disruption in parallel information processing over the whole networks (P< 0.05). The disruption was present in all the functional modules (default mode, executive control, sensorimotor and visual networks) although decreased functional connectivity was observed only within the default mode network. Regional analysis showed that the disease disproportionately weakened nodal efficiency of the default mode components and tended to preferentially affect central or hub-like regions. Intriguingly, the network abnormalities correlated with biochemical hemoglobin and serum calcium levels in the patients. Finally, the functional changes were substantively unchanged after correcting for gray matter atrophy in the patients.

**Conclusion:**

Our findings provide evidence for the disconnection nature of ESRD’s brain and therefore have important implications for understanding the neuropathologic substrate of the disease from disrupted network organization perspective.

## Introduction

The human brain operates essentially as an interconnected network that underscores cognition and behavior [1,2]. Mapping and characterization of such a brain network are thus vital for our understanding of the brain and have attracted great attention in recent years [1,3]. To date, several non-trivial features are consistently revealed in healthy brain, such as small-worldness [4], modularity [5] and hubs [6]. Moreover, accumulating evidence indicates that disturbances in these configurations are largely responsible for cognitive and behavior dysfunction in various brain disorders [7,8], therefore hastening conceptual onset of pathoconnectomics [9], the mapping of abnormal brain networks under pathological conditions.

End-stage renal disease (ESRD) is a disease characterized by multi-organ dysfunction. It typically occurs when chronic renal failure progresses to a point where the kidneys are permanently functioning at less than 10% of their capacity [10]. ESRD is often accompanied by central nervous system abnormalities [11] and neurological problems [12]. More importantly, ESRD significantly elevates the risk of developing cognitive impairments [13–15] and leads to an increased death rate [16–18]. Therefore, understanding brain abnormalities in neurologically asymptomatic patients with ESRD is crucially important for the early diagnosis, prognosis improvement and ultimate, reduction of death rate.

Non-invasive neuroimaging techniques provide promising avenues to achieve these goals and have been increasingly applied to this disease. For example, diffusion tensor imaging and structural MRI studies have found that patients with ESRD exhibit abnormal white matter integrity [19–21] and gray matter (GM) atrophy [22], respectively. Additionally, metabolic or functional disturbances are also reported in the disease by single photon emission tomography, magnetic resonance spectroscopy and arterial spin-labeling (ASL) studies [22–24]. More recently, resting-state functional magnetic resonance imaging (R-fMRI), a technique to measure spontaneous brain activity, is utilized to study intrinsic functional architecture in ESRD. With this technique, decreased regional homogeneity [25] and functional connectivity [26] are found in the default mode network (DMN) in ESRD patients compared to healthy controls (HC). These pioneering studies greatly promote our understanding of the disease. Nevertheless, the neuropathologic substrate underlying the ESRD is far from well-established, particularly at the system-level from a whole-brain network perspective.

To fill the gap, this study systematically investigated ESRD-related alterations in the topological organization of intrinsic whole-brain functional networks in patients with ESRD. For graph-based network studies, the topological analyses can be done at multiple levels, such as whole-brain global topology, regional nodal characteristic and intermediate modular architecture. Different levels can provide unique information regarding how the brain networks adaptively reorganize to respond various brain disorders. To the best of our knowledge, this is the first study to investigate ESRD-related alterations in the topological organization of functional brain networks; we thus aimed to provide comprehensive insights regarding how ESRD affects global, modular and regional network topology. Our results demonstrated abnormal configurations of network organization in all the three levels, which significantly advance our understanding of network-level disturbances in ESRD.

## Materials and Methods

### Participants

This study was approved by the Research Ethics Review Board of the Institute of Mental Health at the Southern Medical University, and written informed consent was obtained from each participant. A total of 25 patients with ESRD (all right-handed) were recruited from the renal transplantation department at Guangdong No. 2 Provincial People's Hospital, Guangzhou, China in this study from August 2011 to July 2012. Exclusion criteria included: (1) psychiatric disorders or major neurologic disorders (e.g., severe head injury, stroke, epilepsy or visible lesions); (2) ischemic diseases including acute ischemic cerebrovascular disease, acute peripheral arterial occlusion, advanced liver or heart failure; (3) asymptomatic coronary ischemia by electrocardiogram testing; (4) a history of diabetes; and (5) substance abuse including drugs, alcohol and cigarettes. Conventional MR images were examined by an experienced radiologist (W. L., with 20 years of experience in neuropathology) who was blinded to whether the images were from the patient or control group. Three patients were excluded due to abnormal hyper-intensities in their T2-FLAIR MR images. Therefore, the final study population included 22 patients with ESRD (16 males, 6 females; mean age 38 ± 10.5 years, range 18–61 years).

All the patients completed biochemical tests, which included serum creatinine, urea level, hemoglobin level, cholesterol level, serum albumin level, serum kalium and serum calcium within 24 hours before the MR imaging. In our study, none of the patients were on erythropoiesis-stimulating agents and none of them were treated with vitamin D, calcitriol and/or phosphorus-chelating agents. We did not check the serum PTH level for the ESRD patients. The serum calcium levels were corrected with serum albumin levels using the Payne's formula [27].The dialysis modality and duration (7.4 ± 2.2 months) were also recorded from the patients’ medical history. Out of the 22 patients with ESRD, twenty had hypertension, and six had hyperlipidemia. In this study, all the patients underwent a neuropsychological test involving a mini-mental state examination (MMSE) [28] and scored > = 28, which indicated their relatively intact global neurocognition.

Twenty-nine age- and gender-matched HCs (all right handed; 19 males, 10 females; mean age 42.1 ± 8.4years, range 28–61years) were recruited from the local community. All the HCs had normal renal function as determined by no abnormal findings in the abdominal MR imaging and had no physical diseases or history of psychiatric or neurologic diseases. All the demographic and clinical data are summarized in Table 1.

**Table 1:** Demographics and clinical characteristics of all participants.

	ESRD (n = 22)	HC (n = 29)	P-value
Gender (M/F)	16/6	19/10	0.583^a^
Age (yrs)	38 ± 10.5 (18–61)	42.1 ± 8.4 (28–61)	0.127^b^
Education level (yrs)	11.4 ± 3.3 (4–16)	12.5 ± 3.4 (6–19)	0.245^b^
Blood systolic pressure	155.3 ± 18.3 (102–190)	110.9± 15.1 (90–145)	<0.001^b^
Blood diastolic pressure	90.1 ± 7.7 (70–100)	75.5 ± 6.7 (60–85)	<0.001^b^
Dialysis duration (mths)	7.4 ±2.2 (2–10)		
Cholesterol (mmol/L)	4.9 ± 1.4 (3.7–9.9)		
Serum calciumc	9.0 ±0.9 (6.8–10.2)		
Serum kalium (mmol/L)^c^	4.0 ±0.6(2.8–5.5)		
Hemoglobin (g/L)c	95.4±22.6 (53–147)		
Serum creatinine (μmol/L)^c^	1005.3± 227.6 (672–1357)		
Blood urea nitrogen (mmol/L)^c^	22.9±7.2 (12.3–36.5)		

Values are represented as mean ± SD (min—max). ESRD, end-stage renal disease; HC, healthy control.

^a^The P-value was obtained by chi-square test.

^b^The P-value was obtained by two-side two-sample t test.

^c^Data were missing for six patients.

### Data Acquisition

All participants were scanned using a 1.5-T MR scanner (Achieva Nova-Dual; Philips, Best, the Netherlands) at the Department of Medical Imaging, Guangdong No. 2 Provincial People’s Hospital. The conventional imaging sequences, which included T1-weighted images and T2-FLAIR images, were obtained for each participant to detect clinically silent lesions. During the R-fMRI data acquisition, the participants were asked to lie quietly with their eyes closed and to not think of anything specific while in the scanner. The scan lasted 8 minutes, and 160 volumes were obtained for each participant. The R-fMRI acquisition parameters were as follows: 33 axial slices; repetition time (TR) = 3,000 ms; echo time (TE) = 50 ms; flip angle = 90°; slice thickness = 4.5 mm; no gap; matrix = 128×128; and field of view (FOV) = 230×230 mm^2^. After the examination, all the participants were asked questions to verify the degree of their cooperation. Additionally, individual high-resolution anatomical images were also acquired using a T1-weighted three-dimensional volumetric magnetization-prepared rapidly acquired gradient-echo sequence: 160 axial slices; TR = 25 ms; TE = 4.1 ms; FA = 30°; slice thickness = 1.0 mm; no gap; matrix = 256×256; and FOV = 230×230 mm^2^.

### Data Preprocessing

The data preprocessing was performed using the SPM8 package (http://www.fil.ion.ucl.ac. uk/spm/software/spm8)and included the following: i) removal of the first five volumes to allow T1 equilibration; ii) realignment to correct for spatial displacements due to head motion; iii) spatial normalization into the Montreal Neurological Institute space; iv) removal of linear trend; v) temporal band-pass filtering (0.01–0.1 Hz); and vi) regression of several nuisance signals of white matter signal, cerebrospinal fluid signal and head-motion profiles.

Recent studies indicate that there still are residual head motion effects on intrinsic functional connectivity and network topology even after typical realignment and regression procedures [29, 30]. To minimize the head motion effects in the current study, we first excluded participants with excessive head motion (>1.5 mm or > 1.5° in any direction). We then examined several summary measures of head motion profiles including the maximum, root mean square and mean frame-wise displacement and found no significant between-group differences (all *P*s > 0.15). Furthermore, instead of six head-motion parameters, we used24 head-motion parameters in the regression model [31] as proposed by a recent study [32], which is an efficiency strategy to control for head motion effects. Finally, we treated all the summary head-motion measures as covariates for the group-level comparisons [33]. With these strategies, we believe that head-motion effects were mitigated as much as possible for the current data.

### Individual-level correlation matrix

We constructed individual brain networks similarly to previous studies [34, 35]. Briefly, the cerebrum were first parceled into 1,024 equal-sized regions of interest (ROIs) [36]. This random parcellation method ensures higher functional homogeneity within the ROIs compared with anatomically defined atlases [37], and the spatial scale (i.e., 1,024 ROIs) is reasonable for an exploratory study of network properties [38]. Notably, the ROIs did not cover the cerebellum and were restricted within a GM mask based on the probability map in SPM8 (threshold = 0.2). We then calculated the Pearson correlation coefficients of the mean regional time series between any pair of ROIs, which generated a 1,024×1,024 connectivity matrix for each participant. To further de-noise spurious interregional correlations, only those correlations whose corresponding *P*-values passed through a statistical threshold (*P*< 0.05, Bonferroni-corrected over connections) were retained. Such a significance level-based thresholding procedure effectively avoids erroneous evaluations of network topology [39]. Notably, negative correlations were also excluded in this study due to their ambiguous interpretation [40–42] and detrimental effects on test-retest reliability [43]. Finally, a sparse, positive and weighted network was obtained for each participant.

### Group-level correlation matrix

All the individual sparse, positive and weighted correlation matrices of the control group were first converted to z-value matrices using a fisher r-to-z transformation to improve normality and then averaged across participants. The resultant z-value matrix was further inversely transformed to an r-value matrix, from which a backbone network was extracted to capture its multiscale structures using a nonparametric sparsification method [44]. The backbone network was subsequently used to identify modular architecture that serves as a reference for between-group comparisons in intra- and inter-module connectivity and intra-module topological analyses.

### Network Analysis

For the constructed brain networks, we calculated several graph-based metrics to characterize their topological organization from different levels: global small-world parameters (global efficiency and local efficiency), intermediate modular composition and local nodal efficiency. We briefly explain these metrics below in the context of a weighted network ***G*** with ***N*** nodes and ***K*** edges. Further details and interpretations of these network measures are described elsewhere [45].

### Small-world parameters

Efficiency is a biologically relevant metric to describe brain networks from the perspective of parallel information flow [46, 47] and can be calculated at both the global and local levels. Mathematically, the global efficiency is defined as:

$$E_{\mathrm{glob}}\left(G\right)=\frac{1}{N\left(N-1\right)}\sum_{i\neq j\in G}\frac{1}{d_{ij}}$$ (1)

where *d*_*ij*_ is the shortest path length between node *i* and node *j* in ***G*** and is calculated as the smallest sum of the edge lengths throughout all of the possible paths from node *i* and node *j*. The length of an edge was designated as the reciprocal of the edge weight (i.e., correlation coefficient), which can be interpreted as a functional distance that a high correlation coefficient corresponds to a short functional distance. Global efficiency measures the ability of parallel information transmission over the network. The local efficiency of ***G*** is measured as:

$$E_{\mathrm{loc}}\left(G\right)=\frac{1}{N}\sum_{i\in G}E_{\mathrm{glob}}(G_{i})$$ (2)

where *E*_*glob*_(*G*_*i*_) is the global efficiency of *G*_*i*_, the subgraph comprised of the neighbors of the node *i* (i.e., nodes linked directly to node *i*). Local efficiency measures the fault tolerance of the network, indicating the capability of information exchange for each subgraph when the index node is eliminated.

To determine whether the constructed brain networks have a small-world organization, the local efficiency and global efficiency were normalized (i.e., *Ẽ*_*loc*_ and *Ẽ*_*glob*_) by dividing them by the corresponding mean derived from 100 random networks that preserved the same number of nodes, edges and degree distributions as the real brain networks [48,49]. Typically, a network is said to be small-world if it has a normalized local efficiency larger than 1 and a normalized global efficiency approximately equal to 1 [50].

### Modular composition

The modularity *Q(p)* for a given partition *p* of a weighted network is defined as:

$$Q\left(p\right)=\sum_{s=1}^{N_{M}}\left[\frac{w_{s}}{W}-{\left(\frac{W_{s}}{2W}\right)}^{2}\right]$$ (3)

where *N*_*M*_ is the number of modules, *W* is the total weight of the network, *w*_*s*_ is the sum of the connectional weights between all the nodes in module *s* and *W*_*s*_ is the sum of the nodal strength (see below for the definition of nodal strength) in module *s*. Modularity quantifies the difference between the weight of the intra-module links of the actual network and those of random networks in which connections are weighted randomly. The aim of the module identification process is to find a specific partition *p* that yields the largest network modularity. Here, we detected modular structure using a spectral optimization algorithm [51].

### Nodal efficiency

The nodal efficiency of a given node *i* is computed as [46]:

$$E_{\mathrm{nodal}}\left(i\right)=\frac{1}{N-1}\sum_{j\neq i\in G}\frac{1}{d_{\mathrm{ij}}}$$ (4)

Nodal efficiency measures the information propagation ability of a node with the rest of the nodes in the network. A node with a high nodal efficiency indicates high information transmission capability with other nodes and therefore can be categorized as a hub.

### Statistical Analysis

Between-group differences in network/connectivity measures were inferred by nonparametric permutation tests. Briefly, for each metric, we initially calculated the between-group difference of the mean values. An empirical distribution of the difference was then obtained by randomly reallocating all of the values into two groups and recomputing the mean differences between the two randomized groups (10,000 permutations). The 95th percentile points of the empirical distribution were used as critical values in a one-tailed test of whether the observed group differences could occur by chance.

A partial correlation analysis was used to assess the relationships between network metrics and clinical variables (dialysis duration, calcium level, kalium level, hemoglobin level, creatinine level and urea level) in the ESRD group after controlling for age, gender and summary measures of head motion.

### Brain Visualization

The brain results were visualized either in surface space using the BrainNet viewer [52] or in volume space using MRIcron (http://www.mccauslandcenter.sc.edu/mricro/mricron/install.html).

## Results

### Demographic and Clinical Characteristics

There were no significant differences in gender (*P* = 0.583), age (*P* = 0.127) or education level (*P* = 0.245) between the ESRD and HC groups. Significantly lower blood systolic pressure and diastolic pressure were observed in the patients compared with the HCs (both *P*s < 0.001).The mean duration of hemodialysis for the patients was 7.4±2.2 months. The mean calcium, kalium, hemoglobin, creatinine, urea and cholesterol levels for the patients were 9.0±0.9, 4.03±0.62 mmol/L, 95.4±22.6 g/L, 1005.3±227.6 μmol/L 22.9±7.2 mmol/L and 4.9±1.4 mmol/L, respectively (Table 1).

### Global Network Organization

**Fig 1:**
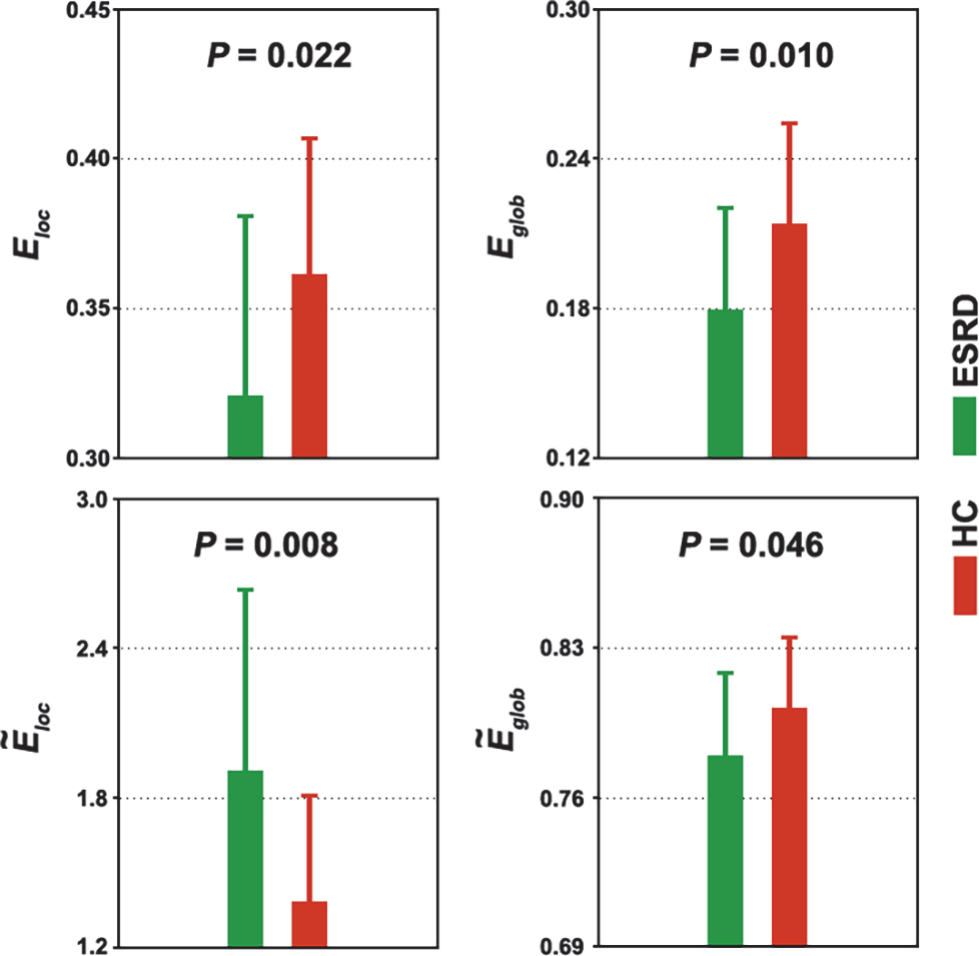
Between-group differences in whole-brain topology. Error bars denote standard deviations. ESRD, end-stage renal disease; HC, healthy control; *E*_*loc*_, local efficiency; *E*_*glob*_, global efficiency; *Ẽ*_*loc*_, normalized local efficiency; *Ẽ*_*glob*_, normalized global efficiency.

We constructed individual functional brain networks by calculating interregional functional connectivity among 1,024 ROIs. After applying a significance-based threshold, the number of survived connections and their associated weights showed no significant between-group differences (all *P*s > 0.05). A further graph-based network efficiency analysis revealed that all the networks obeyed small-world organizations characterized by larger local efficiency but approximately equal global efficiency relative to matched random networks (HC: *Ẽ*_*loc*_ = 1.44 ± 0.47, *Ẽ*_*glob*_ = 0.82 ± 0.03; ESRD: *Ẽ*_*loc*_ = 1.79 ± 0.79, *Ẽ*_*glob*_ = 0.80 ± 0.04). Nevertheless, statistical comparisons revealed significant differences in the quantitative network measures between the two groups. Compared to the HC group, the ESRD patients showed significantly decreased local efficiency (*P* = 0.022) and global efficiency (*P* = 0.010) in their functional brain networks (Fig. 1). When normalized by random networks, local efficiency was increased (*P* = 0.008) and global efficiency (*P* = 0.046) was decreased in the ESRD patients compared to the HCs (Fig. 1).

### Modular Brain Organization

**Fig 2:**
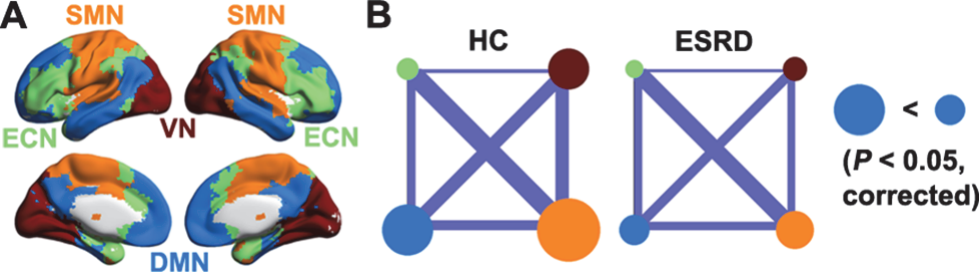
Between-group differences in intra- and inter-module connectivity. A, four modules were identified in group-level functional network of healthy controls, including default mode network (DMN), executive control network (ECN), sensorimotor network (SMN) and visual network (VN). B, patients with ESRD only showed decreased functional connectivity within the DMN. Node size and line width are in proportion to mean intra- and inter-module connectivity strength, respectively. ESRD, end-stage renal disease; HC, healthy control.

Four functional modules were identified in the mean network of the HC group (*Q* = 0.531, *P*< 10^−3^) including the DMN (317 ROIs), executive control network (ECN, 250 ROIs), sensorimotor network (SMN, 289 ROIs) and visual network (VN, 168 ROIs) (Fig. 2A). Under the framework of this modular architecture, we contrasted the intra- and inter-module connectivity strength between the ESRD patients and the HCs. Compared with the HC group, the patients with ESRD showed significantly (*P*< 0.05, False Discovery Rate corrected) decreased functional connectivity within the DMN (Fig. 2). There were no connectivity differences within other modules or between any pair of modules.

**Fig 3:**
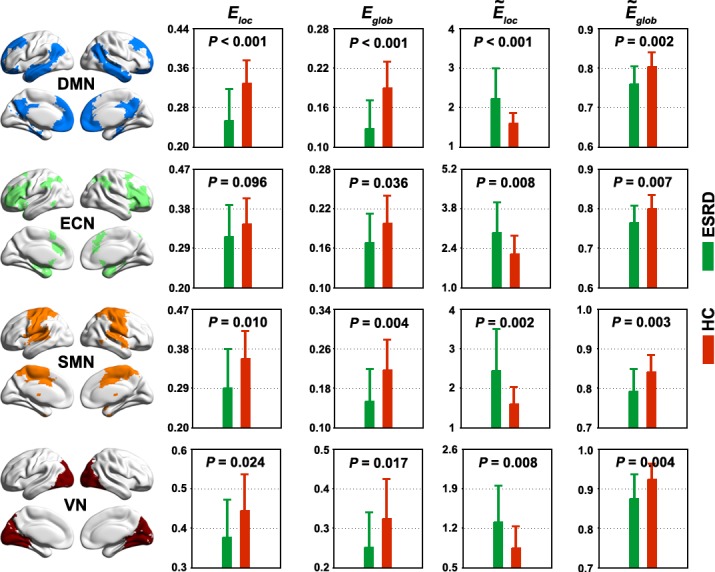
Between-group differences in module-level topology. Each functional module exhibited altered network organization in patients with ESRD compared to HCs in extremely similar patterns to those observed for the whole-brain networks (Fig. 1). Error bars denote standard deviations. ESRD, end-stage renal disease; HC, healthy control; *E*_*loc*_, local efficiency; *E*_*glob*_, global efficiency; *Ẽ*_*loc*_, normalized local efficiency; *Ẽ*_*glob*_, normalized global efficiency; DMN, default mode network; ECN, executive control network; SMN, sensorimotor network; VN, visual network.

We further studied the topological architecture of each module. The results showed that all four modules exhibited significantly different topological organizations between the ESRD and the HC groups in highly similar patterns to those observed for the whole-brain network (Fig. 3).

### Regional Network Organization

**Fig 4:**
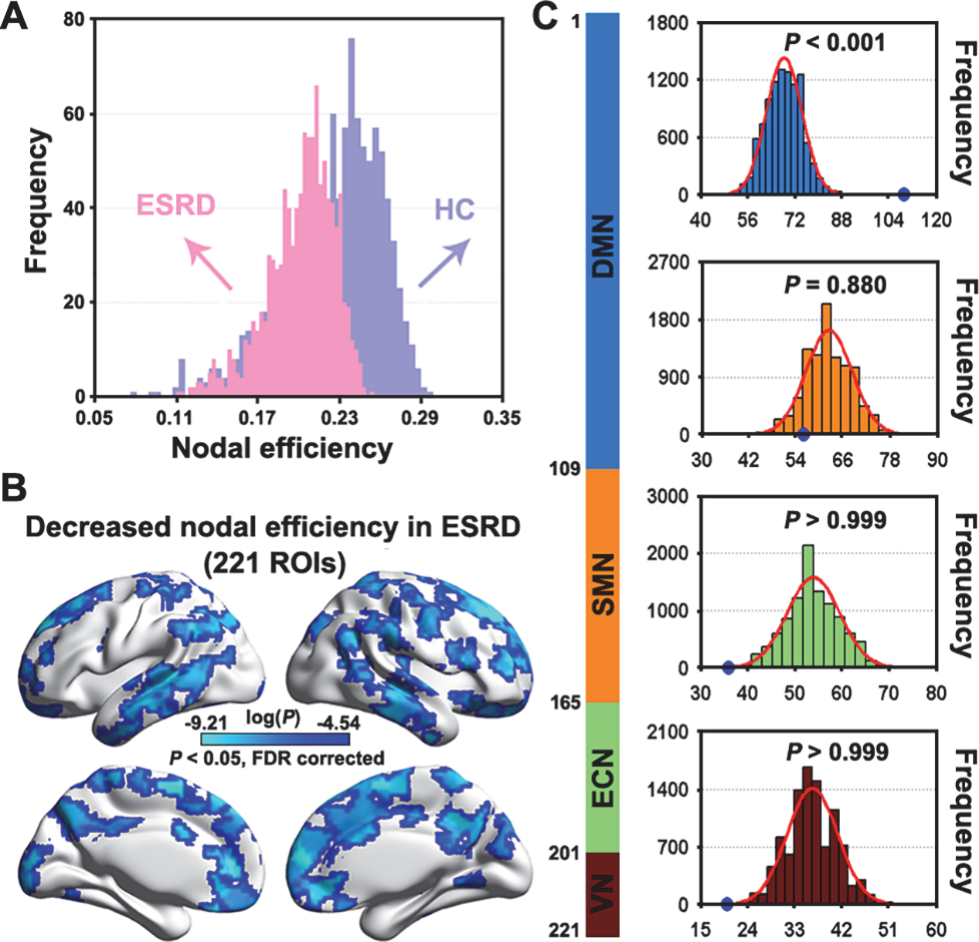
Between-group differences in regional nodal efficiency. A, histogram of mean nodal efficiency showed an evident shift towards overall decrease in patients with ESRD compared to HCs. B, statistical analysis revealed that the global decrease was driven by numerous regions that predominantly located in lateral and medial prefrontal cortex, motor cortex, posterior parietal cortex, lateral temporal lobe and occipital regions. C, empirical frequency distributions of 221 randomly selected ROIs (10,000 times) in each of the four modules in Fig. 2A. ESRD, end-stage renal disease; HC, healthy control; ROIs, regions of interest; DMN, default mode network; ECN, executive control network; SMN, sensorimotor network; VN, visual network.

Compared with the HCs, the ESRD patients showed an overall decrease in the mean nodal strength (Fig. 4A). To localize brain regions that drive the global decrease, further node-wise comparisons revealed a total of 221 ROIs that exhibited significantly (*P* < 0.05, False Discovery Rate corrected) decreased nodal efficiency in the patients compared to the HCs (Fig. 4B). These regions enclosed predominately the lateral and medial prefrontal cortex, motor cortex, anterior and middle cingulate gyri, precuneus, angular gurus, superior parietal lobule, lateral temporal lobe and occipital regions, bilaterally. When superimposed on the modular architecture (Fig. 2A), the 221 ROIs were mainly located in the DMN (109/221, 49.3%), followed by the SMN (56/221, 25.3%), ECN (36/221, 16.3%) and VN (20/221, 9.1%). Given the different sizes among modules, we further performed a statistical test to determine whether such a non-uniform distribution occurs by chance. We randomly selected 221 ROIs from the 1,024 ROIs and recorded their distribution over the four modules. This procedure was implemented 10,000 times to generate empirical distributions of frequency. Intriguingly, we found that the ESRD-related ROIs were susceptible to specific modules of the DMN (*P*< 0.001, Fig. 4C).

**Fig 5:**
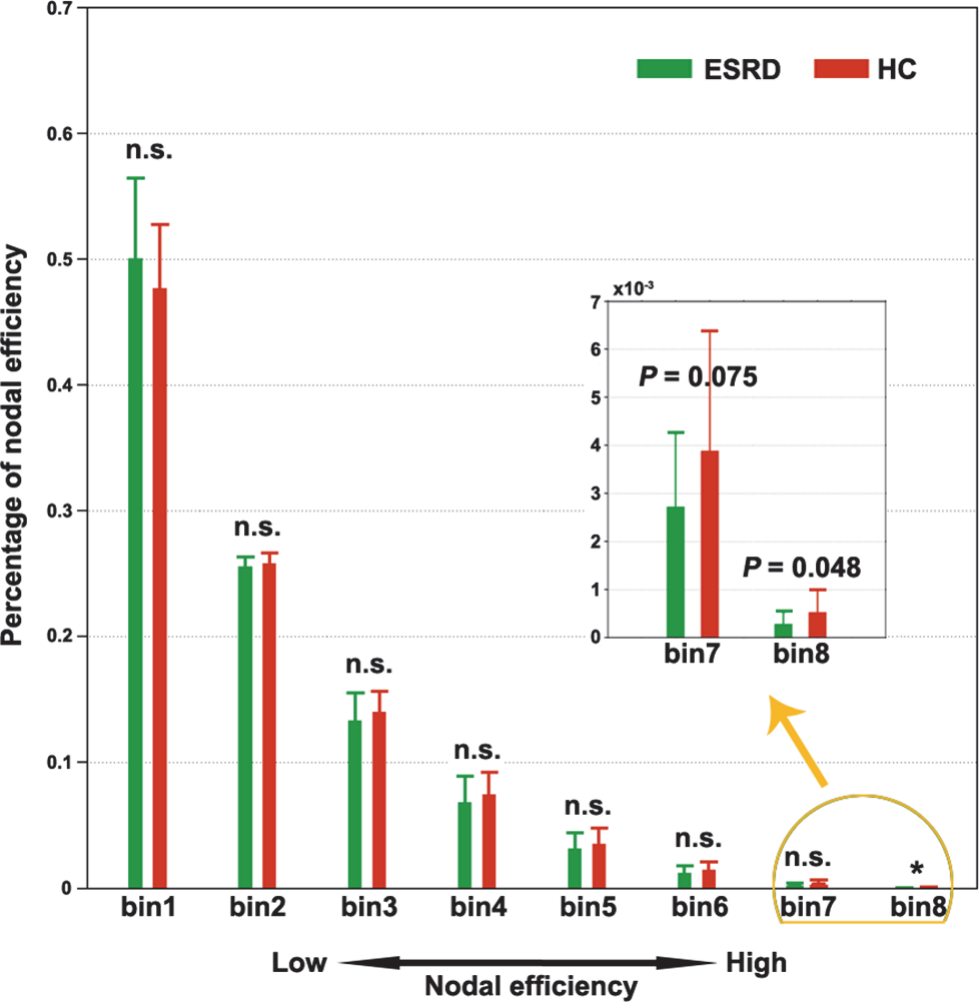
Preferential susceptibility of ESRD to hub-like regions. All the 1,024 regions were equally divided into eight bins (128 nodes in each bin) in terms of their nodal strength for each participant. The percentage of regions falling in each bin was compared and only the seventh and eighth bins (corresponding to large nodal efficiency) were observed to show ESRD-related decreases compared to HCs. n.s., non-significant; ESRD, end-stage renal disease; HC, healthy control.

To further test whether the ESRD affects brain regions differentially, we divided the 1,024 ROIs into 8 equally spaced bins between the minimum and maximum values of nodal efficiency for each participant such that the first bin contained regions with low nodal efficiency (i.e., peripheral regions) and the eighth bin included regions with high nodal efficiency (i.e., central or hub regions). We then compared the percentage of regions that fell into each bin between the two groups. Here, the number of bins is chosen according to the following considerations: 1) 1,024 is divisible by the number of bins; 2) the number of bins should be small enough to avoid a severe multiple comparison issue; and 3) the number of bins should be large enough to group nodes into multiple sets with different nodal efficiency. Based on these criteria, we empirically set the number of bins to 8.The results revealed that the ESRD patients exhibited a significantly decreased percentage in the seventh and eighth bins (Fig. 5), which indicated a preferential susceptibility to hub regions in the disease.

### Relationship between Brain Network Measures and Clinical Variables

**Fig 6:**
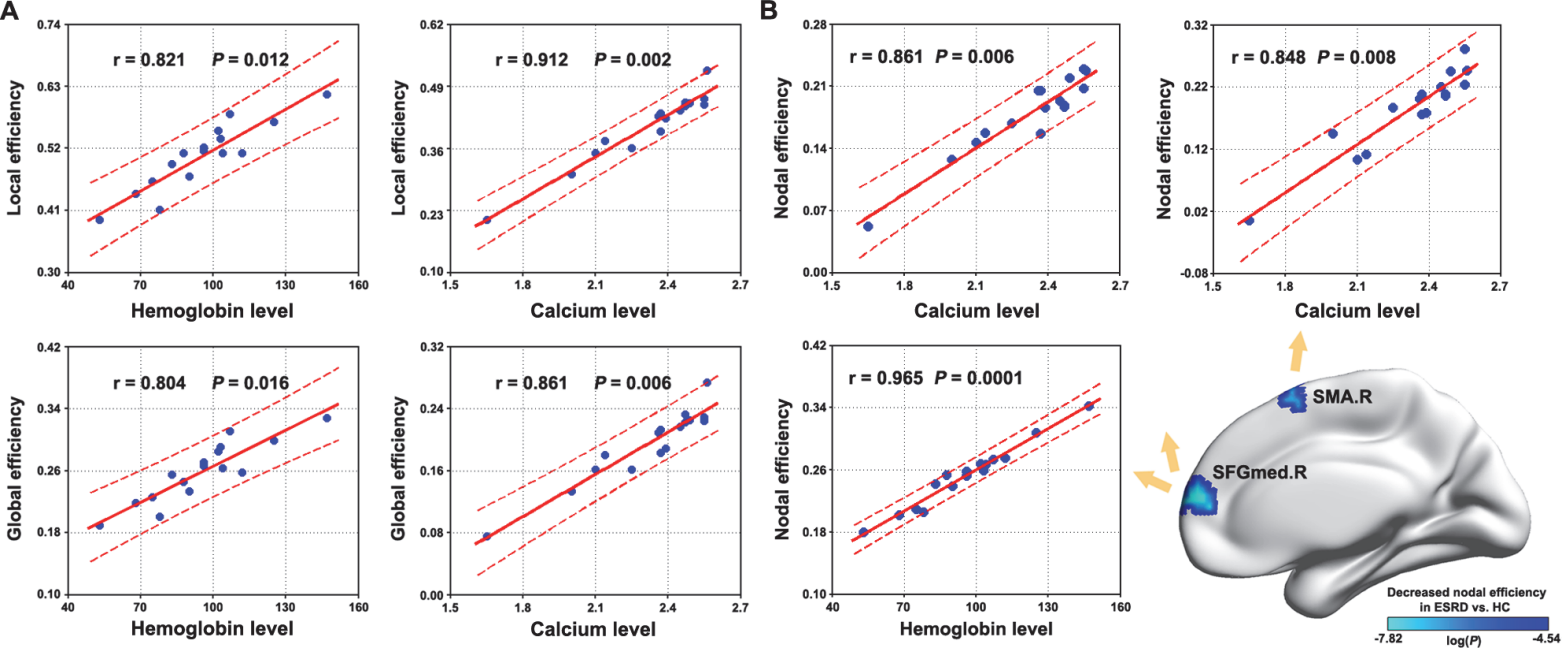
Relationship between network measures and biochemical parameters. Significant correlations were observed for both global network organization and regional nodal efficiency with biochemical parameters in ESRD patients. SMA, supplemental motor area; SFGmed, medial superior frontal gyrus; R, right; ESRD, end-stage renal disease; HC, healthy control.

Significant correlations were observed between the small-world parameters of whole-brain networks and the clinical variables in the patients. Specifically, both local efficiency and global efficiency positively correlated with the hemoglobin level (Fig. 6A). For modules, only the SMN module exhibited significantly positive correlations with the hemoglobin level in the local efficiency and global efficiency and negative correlation with the hemoglobin level in the normalized local efficiency. No correlations were observed for the other three modules (Table 2).Additionally, significantly positive correlations (*P*< 0.05, False Discovery Rate corrected) were observed for the nodal efficiency of the right medial superior frontal gyrus with the hemoglobin level (r = 0.965, *P* = 0.0001) (Fig. 6B).

**Table 2:** Partial correlation coefficients between network metrics and biochemical parameters.

	Network efficiency of whole-brain network			
Biochemical parameters	*E*_*loc*_	*E*_*glob*_	*Ẽ*_*loc*_	*Ẽ*_*glob*_
Hemoglobinlevel	**0.821**	**0.804**	-0.631	0.144
	Network efficiency of DMN module			
Biochemical parameters	*E*_*loc*_	*E*_*glob*_	*Ẽ*_*loc*_	*Ẽ*_*glob*_
Hemoglobinlevel	0.638	0.627	-0.524	0.269
	Network efficiency of ECN module			
Biochemical parameters	*E*_*loc*_	*E*_*glob*_	*Ẽ*_*loc*_	*Ẽ*_*glob*_
Hemoglobinlevel	0.629	0.667	-0.515	0.271
	Network efficiency of SMN module			
Biochemical parameters	*E*_*loc*_	*E*_*glob*_	*Ẽ*_*loc*_	*Ẽ*_*glob*_
Hemoglobinlevel	**0.868**	**0.820**	-**0.604**	0.426
	Network efficiency of VN module			
Biochemical parameters	*E*_*loc*_	*E*_*glob*_	*Ẽ*_*loc*_	*Ẽ*_*glob*_
Hemoglobinlevel	0.687	0.618	-0.504	0.398

The partial correlations were computed with age, gender and summary measures of head motion as the confounding covariates. Values in bold indicate significant correlations (*P*< 0.05). No significant correlations were observed for dialysis duration, kalium level, creatinine level and urea level.

*E*_*loc*_, local efficiency.

DMN, default mode network.

ECN, executive control network.

SMN, sensorimotor network.

VN, visual network.

### GM Volume

**Fig 7:**
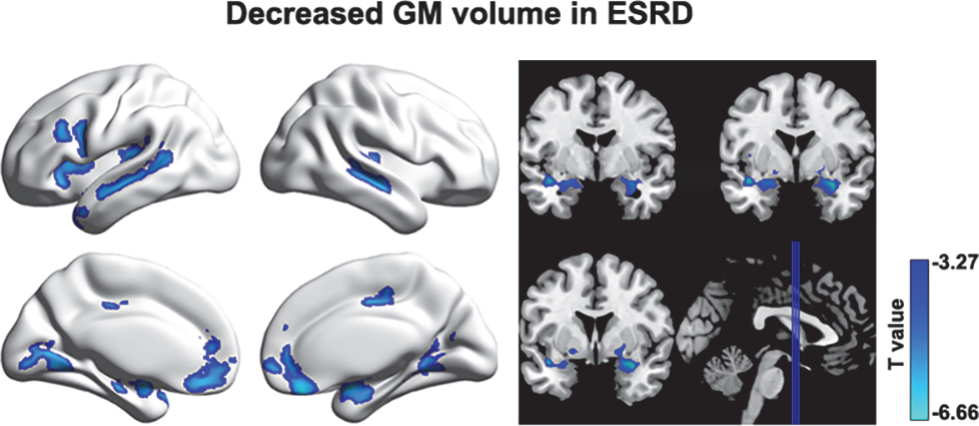
Between-group differences in gray matter volume. Multiple cortical and subcortical regions were identified to show gray matter atrophy in ESRD patients compared to HCs. Of note, no significant correlations were observed between nodal efficiency decreases and GM gray matter atrophy in the patients (P > 0.05, False Discovery Rate corrected). GM, gray matter; ESRD, end-stage renal disease.

A voxel-based morphometry analysis revealed that multiple cortical and subcortical regions exhibited GM atrophy in the patients compared to the HCs (*P*< 0.05, corrected), which included the bilateral medial prefrontal gyrus, anterior cingulate gyrus, superior temporal gyrus, middle temporal gyrus, lingual gyrus, parahippocampa gyrus, putamen, caudate, insula and right middle cingulate gyrus (Fig. 7). There were no significant correlations between functional nodal efficiency and structural mean GM volume for any ROI (*P*> 0.05, False Discovery Rate corrected).

### Reproducibility analyses

**Fig 8:**
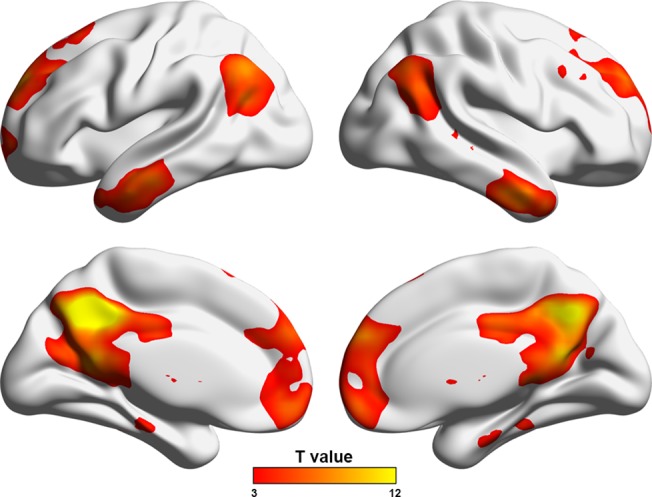
PCC-based functional connectivity.

**Fig 9:**
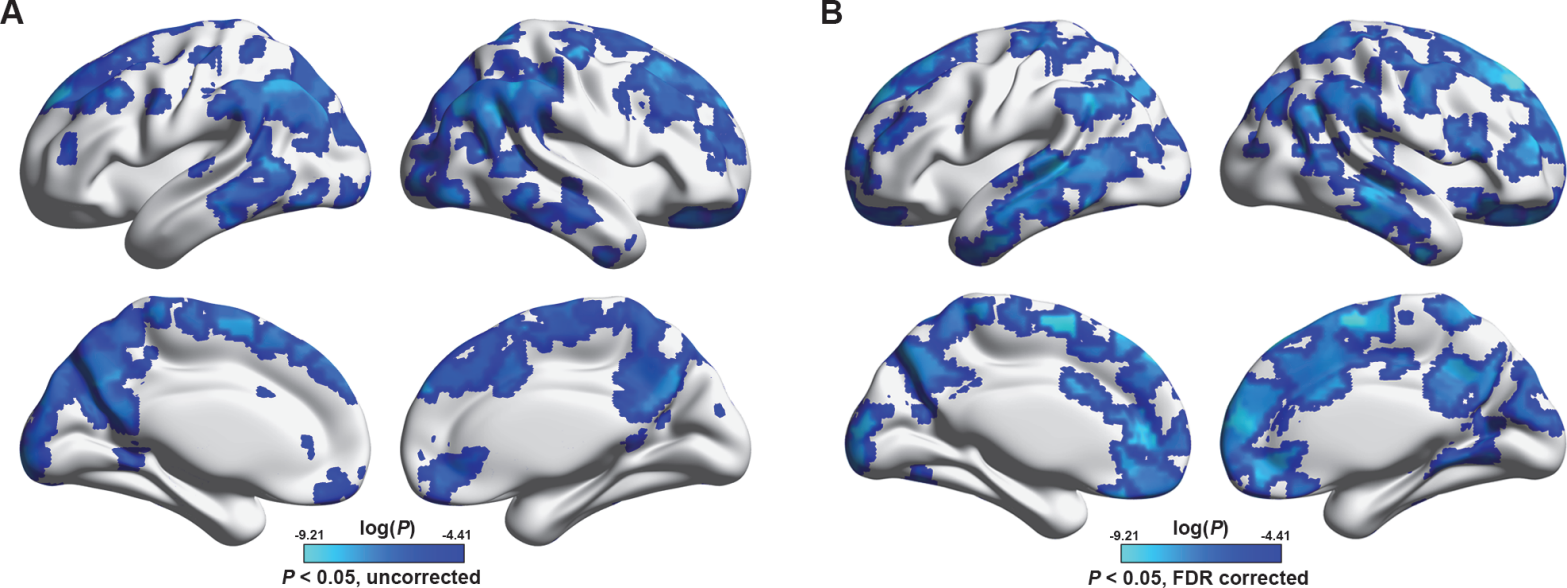
Decreased nodal efficiency in the patients when the global signals were removed (A) or when one patient with the lowest hemoglobin value was excluded (B).

Frist, the current dataset were obtained using a relative low field strength (1.5 T), long TR (3s) and high in-plane resolution (128×128). These may lead to a low SNR for detecting resting-state networks. So we performed a seed-based functional connectivity analysis (seed = the left posterior cingulate cortex, MNI coordinate = [-5 -52 41]) to test whether our data could reproduce the putative default mode network. As shown in the Fig. 8, the mapped connectivity pattern from our data was highly comparable with numerous previous studies (e.g., [53]). Second, the global signal removal is a controversial preprocessing step for resting-state fMRI studies [54–59]. Thus, we re-constructed individual functional brain networks based on the data that underwent global signal removal. Subsequent network analyses showed that the between-group differences became non-significant for global whole-brain network properties (i.e., local and global efficiency and their corresponding normalized versions). As for nodal efficiency, although an overall similar pattern of decreased nodal efficiency was observed in patients with ESRD (Fig. 9A, dice coefficient = 0.45 with those reported in Fig. 4B), the decreases were non-significant after multiple comparison correction. Third, we found that one patient had a very low hemoglobin value (53 g/L), suggesting severe anemia. Therefore, we reanalyzed our data after excluding this patient to test the extent to which our main findings were affected. We found that the results were largely preserved for both whole-brain topology (*P* = 0.022 for local efficiency; *P* = 0.011 for global efficiency; *P* = 0.009 for normalized local efficiency; *P* = 0.047 for normalized global efficiency) and local nodal efficiency (Fig. 9B, dice coefficient = 0.93 with those reported in Fig. 4B).

## Discussion

This study examined the topological organization of functional brain networks in patients with ESRD by combining R-fMRI and graph-based approaches. The results revealed that ESRD patients had abnormally organized brain networks that were manifested at multiple levels of network configurations. Moreover, the altered network architecture was related to the patients’ biochemical indicators. These findings provide direct evidence for network disorganization in ESRD.

The human brain is a complex, interconnected network that continuously integrates information across multiple sensory systems. Numerous studies suggest that the powerful performance of the brain originates from nontrivial small-world organization that confers a capability for both modularprocessing in local neighborhoods (i.e., functional segregation) and integrated processing over distributed brain regions (i.e., functional integration) [1,60,61]. Using computation simulation approaches, Sporns et al. [62] found that small-world topology emerges when networks are evolved into an optimal balance between local specialization and global integration. Here, we also found small-world organizations of functional brain networks in the both groups, consistent with these empirical and computational studies.

Nevertheless, the small-world parameters were quantitatively altered in the patients as characterized by decreased local and global efficiency compared to HCs. Local efficiency is predominantly associated with short-range connections between nearby regions and reflects modular information processing or fault-tolerance of a network [47]. Global efficiency is mainly associated with long-range connections and reflects integrative information processing between and across remote regions of the brain that constitutes the basis of cognitive processing [63]. The observed decreases thus suggest impaired functional segregation and integration in the disease, which presumably are due to disrupted interregional coordination both among local neighbors and across distant regions. This was supported by the finding of decreased nodal efficiency in numerous regions caused by ESRD. When normalized by random networks, local efficiency was increased while global efficiency was decreased in the ESRD patients compared to HCs. This combination jointly suggests a shift towards regular configurations in ESRD’s brain that favor higher modular processing but lower global coordination compared to the small-world organization. Since the small-world model reflects an optimal balance between local specialization and global integration, these results indicate a disruption in the normal balance of functional brain networks of ESRD. It should be noted that these abnormalities were sensitive to the global signal removal, indicating that the findings should be interpreted with caution.

We further examined the small-world parameters of each functional module motived by the fact that different modules possess unique organizations [64, 65]. Four modules were identified in the HC group, largely consistent with those in a previous study [66]. In the modular framework, all the modules were aberrantly organized in ESRD in a similar manner to that of whole-brain networks. These findings indicate that as a general change in ESRD, disrupted balances between local specialization and global integration are involved in all functional systems. This is consistent with previous findings that ESRD is associated with diffused gray matter atrophy [67] and white matter damage [19, 20] over the cerebral mantle. Before any overt neurological manifestation, patients with ESRD are often accompanied with various cognitive deficits, such as attention, processing speed [68], executive function [13], motor function [69] and memory [70]. We speculate that the generality of cognitive disturbances in ESRD may (at least partly) be attributable to widespread disruptions in network organization. Notably, the patients in this study were cognitively intact globally (MMSE > = 28). Therefore, altered network organization may be an early predictive sign of cognitive dysfunction in the disease. Future studies are required to determine the dynamic reconfiguration of network organization with the progress of the disease.

We found positive correlations between decreased efficiency and hemoglobin levels in patients with ESRD. ESRD patients usually have normocytic anemia and other complications of malnutrition and/or protein-energy wasting with different pathophysiology. Previous studies have shown that chronic malnutrition can result in insufficient nutrient supply to the brain that subsequently triggers a series of problems, including brain tissue hypoxia and blood viscosity reduction and eventual hypoperfusion and/or hypometabolism [71,72].There is also increasing evidence supporting that a long-term hemodialysis could lead to remarkable cerebral abnormalities of oxygenation [73] and cerebral blood flow [74–76] in ESRD, which can significantly affect the cerebral circulation and brain function [77–80]. In addition, vascular dementia is reported much more common in ESRD [73,81]. These studies suggest a significant effect of intermittent hemodialysis on cerebral perfusion. Recent studies have highlighted the importance of normal metabolism in establishing and retaining interregional coordination in the brain [66, 82], the basis of cognitive processing [63]. Given that low hemoglobin is associated with poor mental health in ESRD [71,76,83], we speculate that decreased interregional connectivity and therefore decreased network efficiency may contribute to cognitive disturbances in ESRD due to insufficient energy metabolism in their brain caused by low hemoglobin levels. This could be tested by simultaneously recording fMRI and ASL and/or positron emission tomography data in the same cohort of participants. Interestingly, the module-level analysis revealed that only the SMN exhibited significant correlations with the hemoglobin levels, indicating unique contributions of this network to the observed global brain-biochemistry correlations.

We found decreased intra-module functional connectivity for the DMN in the ESRD patients. Previous studies have suggested that the DMN components are structurally connected [84, 85] and show coherent brain activity in both humans [53,86] and non-human animals [87, 88]. Thus, the decrease indicates a weaker coherence or coordination of spontaneous brain activity in the DMN in ESRD. This is consistent with previous reports using regional homogeneity [25] and independent component analysis [26] methods. These studies collectively suggest that decoupling within the DMN is a robust change in ESRD against different analytic approaches.

At nodal level, numerous regions showed decreased nodal efficiency in the ESRD patients, comparable with those showing hypoperfusion [89] or hypometabolism [90] in patients with chronic kidney disease. Intriguingly, we observed that ESRD disproportionately affected the DMN components and preferentially targeted hub regions. This is consistent with the current consensus that hubs are predominantly located in the DMN [91–93]. The DMN is engaged in a wide spectrum of cognitive processing [94] and has high metabolism and oscillation power of spontaneous neural activity [95, 96]. The functional diversity and high-level activity of the DMN inevitably require a high energy intake. However, as we discussed above, patients with ESRD typically suffer from insufficient energy metabolism in their brain, which may disrupt interregional connectivity [66]. Given the high connectivity of the DMN components, it is reasonable to observe the most salient disruptions in these regions. This was further supported by the positive correlation between nodal efficiency of the medial superior frontal gyrus (a key node in the DMN) and hemoglobin levels in the patients. Additionally, we also found widespread gray matter atrophy in the patients with ESRD, which is consistent with a previous morphological study in ESRD [67]. Furthermore, no correlations were detected between gray matter atrophy and nodal efficiency decreases in the patients, suggesting the independence of functional abnormalities from morphological changes. As for structure-function relationship, we found no significant correlations between nodal efficiency and regional gray matter volume, suggesting that functional network abnormalities were largely independent of morphological alterations. Previous studies have shown that regional gray matter morphology is related to local functional organization (e.g., regional functional homogeneity and amplitude of low frequency fluctuation) under both healthy and pathological conditions [97,98], implying a tight coupling between regional brain morphology and local functional architecture. However, in the current study we did not detect significant correlations between regional gray matter volume and nodal efficiency. Presumably, this may be because that instead of quantifying local functional architecture, nodal efficiency reflects the efficiency of interregional information processing for a given region with all the other regions in the brain. Therefore, the lack of significant correlations between regional gray matter volume and nodal efficiency sounds plausible because there is limited evidence for the effects of regional brain morphology on how a region interacts with all the other regions in the brain. Nevertheless, it should be noted that the structure-function relationship is an ongoing filed [99] and is still not fully understood, especially at local brain organization. Future studies are warranted to provide more insights into this issue.

## Limitation

Several issues need to be addressed in future. First, the sample size was relatively small and the MRI scanning parameters were suboptimal in the current study, which could limit the detection of more subtle abnormalities in the disease. Future studies in a large cohort of participants are needed to reproduce the current findings by using more advanced techniques and optimized parameters. Second, there are numerous alternative methodologies used during complex brain network studies, such as brain parcellation schemes [37,100], functional connectivity measures [101,102], thresholding procedures [39, 103] and null models [104–106]. How these factors affect the current findings should be tested in the future. Third, due to the cross-sectional design of the current study, we cannot address how functional brain networks adaptively reorganize in response to the progress of ESRD. Future longitudinal studies would provide insights into this issue. Fourth, consist with a recent R-fMRI study [26], several comorbidities associated with ESRD (e.g., anemia, hypertension and hyperlipidemia) existed for the patients in the current study. Therefore, it is likely that the observed network abnormalities are a common consequence of both ESRD and these comorbidities. Future studies are needed to help clarify this issue by recruiting more homogeneous and purer samples. Finally, accumulating evidence suggests that functional brain networks are largely shaped by underlying structural pathways [107, 108]. Although a recent diffusion tensor imaging study demonstrated abnormalities in several specific neural tracts in neurologically asymptomatic patients with ESRD [21], whole-brain structural networks still remain to be elucidated in ESRD to examine whether the functional brain network changes observed here have a structural substrate.

## Conclusion

The current study demonstrates the disconnectivity nature of ESRD’s brain that is evident in global network organization, intra-module functional integration and regional nodal efficiency. Moreover, the disconnectivity is related to biochemical parameters in the patients. These findings provide novel insights in understanding ESRD from large-scale network perspective.
